# MiR-450a-5p Inhibits Gastric Cancer Cell Proliferation, Migration, and Invasion and Promotes Apoptosis *via* Targeting CREB1 and Inhibiting AKT/GSK-3β Signaling Pathway

**DOI:** 10.3389/fonc.2021.633366

**Published:** 2021-03-29

**Authors:** Ya-Jun Zhao, Jun Zhang, Yong-Cang Wang, Liang Wang, Xin-Yang He

**Affiliations:** ^1^ Department of Gastrointestinal Oncology Surgery, The First Affiliated Hospital of University of Science and Technology of China, Division of Life Sciences and Medicine, University of Science and Technology of China, Hefei, China; ^2^ Center for Diagnostic Pathology, The First Affiliated Hospital of University of Science and Technology of China, Division of Life Sciences and Medicine, University of Science and Technology of China, Hefei, China; ^3^ Department of General Surgery, The First Affiliated Hospital of University of Science and Technology of China, Division of Life Sciences and Medicine, University of Science and Technology of China, Hefei, China

**Keywords:** gastric cancer, proliferation, apoptosis, CREB1, AKT/GSK-3β signaling pathway, miR-450a-5p

## Abstract

Gastric cancer seriously affects human health and research on gastric cancer is attracting more and more attentions. In recent years, molecular targets have become the research focus. Accumulating evidence indicates that miR-450a-5p plays a critical role in cancer progression. However, the biological role of miR-450a-5p in gastric carcinogenesis remains largely unknown. In this study, we explore the effects and mechanisms of miR-450a-5p on the development and progression of gastric cancer. We used gain-of-function approaches to investigate the role of miR-450a-5p on gastric cancer cell proliferation, migration, invasion, and apoptosis using biological and molecular techniques including real-time quantitative PCR (RT-qPCR), CCK-8, colony formation, flow cytometry, Western blot, wound healing, transwell chamber, dual luciferase reporter, and tumor xenograft mouse model. We found that gastric cancer cells have low expression of miR-450a-5p and overexpression of miR-450a-5p inhibited gastric cancer cell proliferation, migration and invasion, and induced apoptosis *in vitro*. Moreover, we demonstrated that ectopic expression of miR-450a-5p inhibited gastric cancer growth *in vivo*. At the molecular level, overexpression of miR-450a-5p significantly increased the expression of pro-apoptotic proteins, including caspase-3, caspase-9, and Bax, and inhibited the expression of anti-apoptotic protein Bcl-2. Luciferase reporter experiment suggested that camp response element binding protein 1 (CREB1) had a negative correlation with miR-450a-5p expression, and knockdown of CREB1 alleviated gastric cancer growth. Furthermore, we also found that miR-450a-5p inhibited the activation of AKT/GSK-3β signaling pathway to inhibit the progression of gastric cancer. Collectively, miR-450a-5p repressed gastric cancer cell proliferation, migration and invasion and induced apoptosis through targeting CREB1 by inhibiting AKT/GSK-3β signaling pathway. MiR-450a-5p could be a novel molecular target for the treatment of gastric cancer.

## Introduction

Human gastric cancer is one of the common malignancies around the world. Approximately 1,000,000 cases of gastric cancer are diagnosed and 783,000 deaths occur in 2018 (1). Different treatment strategies, including surgery, chemotherapy, and radiation, have achieved remarkable advance. However, the overall therapeutic outcome for advanced disease remains poor (2, 3). Thus, it is critical to better understand the molecular mechanisms and explore new treatment strategies for improving the treatment of gastric cancer.

MicroRNAs (miRNAs) are a kind of endogenous small RNA with a length of about 20–24 nucleotides, that have a variety of important regulatory roles in cells. Previous studies showed that miRNAs are involved in regulating the development of gastric cancer. MiR-4317 inhibits cell proliferation and blocks the conversion of S-G2/M phase in gastric cancer cells, and is a promising therapeutic molecular target (4). MiR-455 suppresses cell proliferation and migration by inhibiting EGFR, acting as a potential target for treatment of gastric cancer (5). MiR-582-5p inhibits cell proliferation and promotes apoptosis by regulating AKT3 (6). Other studies have also shown the inhibitory effect of miRNAs on the progression and development of gastric cancer, including miR-744, miR-140-5p, miR-181a, miR-182, miR-802, miR-21, and miR-149 (7–14). On the other hand, miR-450, as a novel miRNA, functions as a tumor suppressor in colorectal cancer (15). However, its specific role in gastric cancer and the underlying mechanism remain unknown.

The role of CAMP-response element binding protein (CREB1) has been reported previously. For instance, aberrant expression of CREB1 affects proliferation and invasion of cancer cells, and has been highlighted in various pathologies (16, 17).Yang et al. demonstrated that CREB1 was a target of miR-134-5p and affected infarct-induced cardiomyocyte apoptosis (18).

In the present study, we found that gastric cancer cells have low expression of miR-450a-5p. Overexpression of miR-450a-5p inhibited cell proliferation, migration, and invasion and induced cell apoptosis in gastric cancer cells both *in vitro* and *in vivo*. Furthermore, we found that CREB1 is targeted by miR-450a-5p and mediated the anti-tumor effects of miR-450a-5p by inhibiting AKT/GSK-3β signaling pathway. Our study indicated that miR-450a-5p could be a potential molecular target for the treatment of gastric cancer.

## Materials and Methods

### Cell Culture

The human gastric cancer cell lines (BGC-823, SGC7901) and human gastric epithelial cell (GES-1) were purchased from the Cell Bank of the Chinese Academy of Science (Shanghai, China). Cells were maintained in Dulbecco’s modified Eagle’s Medium (DMEM) containing 10% fetal bovine serum (FBS, Gibco, NY, USA) in a humidified chamber with 5% CO_2_ atmosphere at 37°C.

### RNA Extraction and RT-qPCR Assay

RNA was isolated using TRIzol (Invitrogen, CA, USA) reagent according to the manufacturer’s protocols. RNAs were reverse transcribed to cDNA by using PrimeScript RT Master Mix (Takara, Dalian, China) following the manufacturer’s protocol. PCR amplification was conducted with the SYBR Premix Ex TaqTM Kit (Takara, Dalian, China). GAPDH and U6 were used to normalize the expression. The relative expression was calculated using the 2^−ΔΔCt^ method.

### Oligonucleotides and Transfection

The plasmid carrying the CREB1 CDS domain was used to overexpress the CREB1 in gastric cancer cells and a pcDNA (pcDNA3.1) vector was used as a negative control (GenePharma, Guangzhou, China). The cDNA encoding CREB1 CDS domain was amplified by PCR, and then sub-cloned into the pcDNA3.1 vector (Invitrogen, CA, USA) to obtain the pcDNA-CREB1. Short hairpin RNA (shRNA) against CREB1(sh-CREB1), their corresponding negative control (sh-NC), miR-450a-5p mimic, and the negative control (NC) were synthesized and purchased from GenePharma (Guangzhou, China). The transfection was performed by using Lipofectamine 3000 Reagent (Life Technologies, Carlsbad, CA, USA).

### Cell Proliferation Assay

Cell Counting Kit-8 (CCK-8) and colony formation assays were performed to examine gastric cancer cell proliferation. In brief, BGC-823 or SGC7901 cells (1 × 10^4^/well) were seeded in 96-well plates and incubated for 0, 24, 48, and 72 h. Then BGC-823 or SGC7901 cells were incubated with 10 μl CCK-8 solution for 4 h at 37°C. The optical density (OD) was recorded at 490 nm using a microplate reader (Multiskan MK3, Thermo Scientific, USA). For colony formation assay, BGC-823 and SGC7901 cells (2 × 10^4^ cells/well) were seeded in 24-well plates. After incubation for 12 days, the cells were immobilized with paraformaldehyde for 30 min, and stained with 10% crystal violet for another 30 min. Colonies were counted and photographed with a light microscope (Olympus, Tokyo, Japan).

### Cell Apoptosis Assay

Flow cytometry was conducted to investigate the gastric cancer cell apoptosis. Cells (1×10^4^ cells/well) were seeded in six-well plates and culture for 48 h. Thereafter, cells were washed using PBS, treated with 5 µl of annexin V-FITC and 5 µl of PI in the dark for 15 min at room temperature. Then apoptotic cells were detected through a FACSCalibur Flow Cytometry (BD Biosciences, CA, USA).

### Cell Migration and Invasion Assays

Wound healing and transwell chamber (5-μm pore size, Costar, Cambridge, MA, USA) assays were conducted to examine gastric cancer cell migration and invasion. For wound healing assay, cells were seeded into six-well plates. The supernatant fluid was removed when BGC-823 or SGC7901 cells were highly confluent (> 90%). Scratches were made using a sterile pipette tip, with the scratch width remaining the same. After continuous culture for 48 h, the width of the scratch was photographed and recorded under a microscope (100×).The distance was assessed by ImageJ software (ImageJ Software Inc., MD, USA). Wound healing rate = (scratch width at 0–48 h)/scratch width at 0 h × 100%.

Cell invasion assay was performed using transwells. In short, transfected BGC-823 or SGC7901 cells were added to the upper chamber loaded with Matrigel (Corning, Cambridge, MA)and the bottom of the chamber were supplemented with complete medium containing 1% FBS. 48 h later, cells on the surface of membranes were wiped out. Invaded cells were fixed in 10% formaldehyde, dyed with 0.1% crystal violet and then counted under a light microscope (Olympus, Tokyo, Japan) (200×).

### Dual Luciferase Reporter Assay

A putative 3’-untranslated regions (3’-UTR) of CREB1 was mutated using mutagenesis kit (Promega, USA). Wild type and mutant sequences were amplified and inserted into the vector to construct luciferase reporter plasmids according to the manufacturer’s instructions (Promega, USA). The luciferase activities were detected with the dual luciferase reporter kit (Promega, USA). Luciferase activity was measured by dual-luciferase reporter assay system (Promega) and presented as firefly luciferase intensity normalized to Renilla luciferase activity.

### Western Blotting Analysis

Total proteins in tissues or cells were extracted with RIPA lysate buffer (Beyotime Inc, Shanghai, China). The protein was quantified using BCA protein assay Kit (Thermo Scientific, CA, USA). The protein samples were separated by 12% polyacrylamide gel, which were transferred onto the PVDF membrane, sealed with 5% skim milk powder. The membrane was incubated with the primary antibody at 4°C overnight and then incubated with HRP coupled secondary antibody (Santa Cruz Inc, CA, USA) at room temperature for 1 h. The protein signal was detected by ECL detection reagents (Thermo Scientific, CA, USA) and GAPDH was used as the internal reference.

### Xenograft Tumors in Nude Mice

Female nude mice (6-week-old, 18–22 g) were provided by Nanjing Medical University and housed under germ free conditions. Animal care and use were carried out according to the ethical guidelines by the First Affiliated Hospital of USTC Animal Care and Use Committee and approved by the Ethics Committee of the First Affiliated Hospital. Nude mice were maintained in a 12h light/12 dark cycle in a temperature- and humidity-controlled environment. To detect the effect of miR-450a-5p on tumor growth *in vivo*, BGC-823 cells (1 × 10^6^ cells) were injected subcutaneously into the right axilla of the nude mice. Following a 30-day period, nude mice were sacrificed, and tumors were isolated for further analyses. Note that the tumor volumes were recorded every week and calculated with the formula: volume = 0.5 × length × wide^2^.

### Statistical Analyses

All data are presented as the mean ± SD. Each bar expressed the mean ± SD of three independent experiments. Statistical significance between two or multiple groups was analyzed by t-test or one-way ANOVA using SPSS 17.0 (SPSS Inc, Chicago, IL, USA). Experiments were repeated three times independently. Statistical significance was assumed when *P*< 0.05.

## Results

### Effect of miR-450a-5p on Gastric Cancer Cell Proliferation and Apoptosis

**Figure 1:**
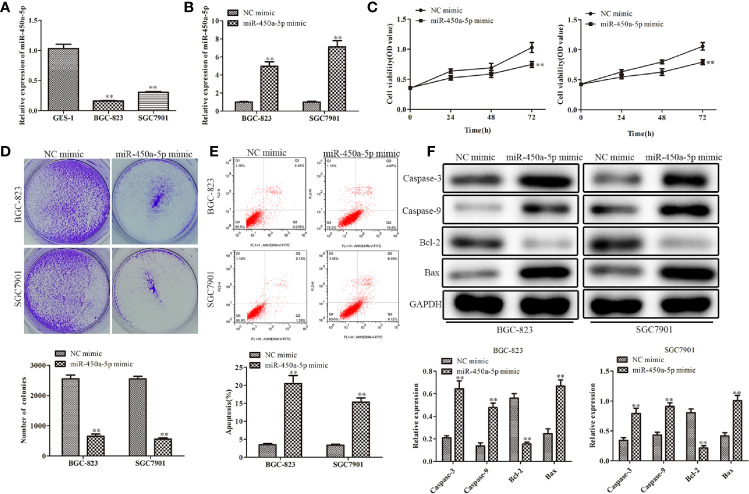
Overexpression of miR-450a-5p inhibits cell proliferation and promotes apoptosis of gastric cancer. **(A)** The expression of miR-450a-5p measured by RT-qPCR in gastric cancer cell lines (BGC-823 and SGC-7901) and a human gastric epithelial cell line (GES-1). ^**^ *P* <0.01 *vs.* GES-1 group. **(B)** The expression of miR-450a-5p assessed by RT-qPCR assay. ^**^ *P* <0.01 *vs.* miR-NC group. **(C–E)** CCK-8, colony formation, and flow cytometry assays detected the cell proliferation and apoptosis in BGC-823 and SGC-7901 cells. ^**^ *P* <0.01 *vs.* miR-NC group. **(F)** Western blot analysis evaluated the expression of apoptosis-associated proteins in BGC-823 and SGC-7901 cells. ^**^ *P* <0.01 *vs.* miR-NC group.

RT-qPCR analysis was employed to examine the expression of miR-450a-5p in gastric cancer cells. As shown in **Figure 1A**, gastric cancer cells (BGC-823 and SGC7901) had low expression of miR-450a-5p compared with the human gastric epithelial cell (GES-1). To further evaluate the roles of miR-450a-5p in the regulation of gastric cancer, miR-450a-5p mimic was transfected into BGC-823 and SGC7901 gastric cancer cells for gain-of-function assays. The efficiency of transfection was validated by RT-qPCR assay (**Figure 1B**). CCK-8 and colony formation assays were performed to evaluate cell viability and proliferation of BGC-823 and SGC7901 cells. The results indicated that the cell viability and number of colonies were significantly decreased in BGC-823 and SGC7901 cells transfected with miR-450a-5p mimic, compared with that in NC group (**Figures 1C, D**). Subsequently, flow cytometry assay was conducted to examine cell apoptosis of these cells. We found that cell apoptosis was significantly increased in miR-450a-5p mimic group, compared with NC group (**Figure 1E**). Consistent with these findings, Western blot analysis revealed that the expression levels of apoptosis-associated proteins, including caspase-3, caspase-9, and Bax, were significantly increased, whereas the anti-apoptotic protein Bcl-2 was decreased in BGC-823 and SGC7901 cells transfected with miR-450a-5p mimic (**Figure 1F**). These results indicated that miR-450a-5p overexpression inhibited cell proliferation and induced cell apoptosis of gastric cancer.

### Overexpression of miR-450a-5p Inhibits Tumor Growth *In Vivo*

**Figure 2:**
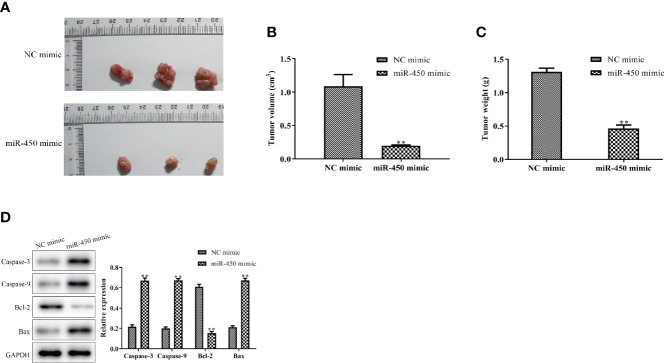
Overexpression of miR-450a-5p inhibits tumor growth. **(A–C)** Tumor phenotype, tumor volume, and weight. **(D)** Western blot analysis on the expression of apoptosis-associated proteins in tumor tissues. ^**^ *P* <0.01 *vs.* miR-NC group.

To confirm whether miR-450a-5p inhibit gastric cancer growth *in vivo*, we generated BGC-823/miR-450a-5p mimic cells and their negative control, then injected them into nude mice to establish tumor xenograft mouse models. Representative images of tumors with miR-450a-5p mimic and NC in the nude mice were shown in **Figure 2A**. Tumor volume and tumor weight were significantly decreased in miR-450a-5p mimic group compared with that in NC group (**Figures 2B, C**). Furthermore, Western blot assay suggested that the expression of apoptosis-associated proteins, including caspase-3, caspase-9, and Bax, were significantly decreased in miR-450a-5p mimic group compared with that in NC group. On the contrary, the expression of Bcl-2 increased in miR-450a-5p mimic group compared with that in NC group (**Figure 2D**). These results indicated that overexpression of miR-450a-5p suppressed gastric cancer growth *in vivo*.

### Overexpression of miR-450a-5p Inhibits Gastric Cancer Cell Migration and Invasion

**Figure 3:**
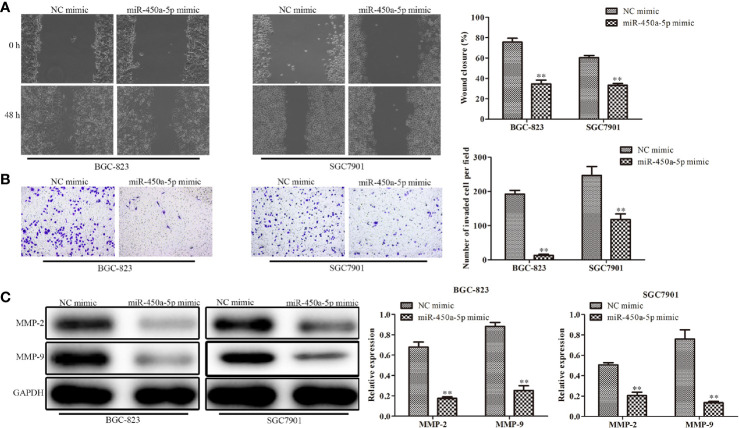
Overexpression of miR-450a-5p inhibits gastric cancer cell migration and invasion. **(A)** Wound healing assay on the cell migration capability of BGC-823 and SGC-7901 gastric cancer cells after transfection of miR-450a-5p mimic. **(B)** Transwell chamber assay on the cell invasion capability of BGC-823 and SGC-7901 gastric cancer cells after transfection of miR-450a-5p mimic. **(C)** Western blot assay on the expression of matrix proteins in BGC-823 and SGC-7901 gastric cancer cells after transfection of miR-450a-5p mimic. ^**^ *P* <0.01 *vs.* miR-NC group.

Cell migration and invasion play important role in the pathogenesis of cancer metastasis. To evaluate the effect of miR-450a-5p on tumor migration and invasion, wound healing assay, and transwell assay were carried out. The results showed that the wound closure rate was significantly decreased in miR-450a-5p-overexpressed BGC-823 and SGC7901 gastric cancer cells, compared with that in NC group (**Figure 3A**). Moreover, the results of transwell invasion assay showed that ectopic expression of miR-450a-5p limited the invasive capability of BGC-823 and SGC7901 cells, compared with that in NC group (**Figure 3B**). Furthermore, Western blot assay suggested that the expression of matrix proteins that are associated with migration- and invasion, such as MMP-2 and MMP-9, were significantly decreased in both BGC-823 and SGC7901 cells transfected with miR-450a-5p mimic, compared with that in NC group (**Figure 3C**). These results suggested that overexpression of miR-450a-5p inhibits migration and invasion of BGC-823 and SGC7901 cells.

### MiR-450a-5p Negatively Regulates CREB1

**Figure 4:**
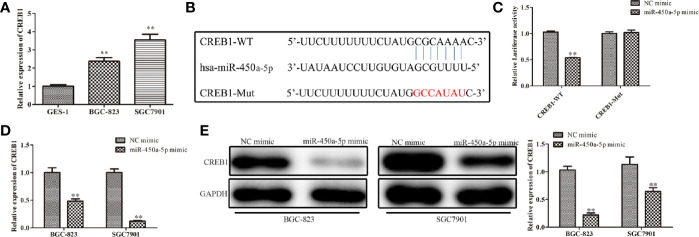
MiR-450a-5p targets and negatively regulates CREB1. **(A)** The expression of CREB1 determined by RT-qPCR in GES-1, BGC-823, and SGC7801 cells. ^*^ *P* <0.05 *vs.* GES-1 group. **(B)** The binding sites between miR-450a-5p and CREB1 were predicted. **(C)** Dual luciferase reporter assay performed in HEK-293T cells to detect the interaction between miR-450a-5p and CREB1. ^**^ *P* <0.01 *vs.* miR-NC group. **(D)** RT-qPCR on the CREB1 expression after transfection of miR-450a-5p mimic. ^**^ *P* <0.01 *vs.* miR-NC group. **(E)** Western blot assay on the CREB1 expression after transfection of miR-450a-5p mimic. ^**^ *P* <0.01 *vs.* miR-NC group.

To study the possible targets of miR-450a-5p involved in the occurrence of gastric cancer, TargetScan was carried out. Thenbsp;result predicted that CREB1 was a potential candidate of miR-450a-5p (**Figure 4A**). The expression of CREB1 in gastric cancer cells was investigated. It was found that BGC-823 and SGC7901 gastric cancer cells have overexpression of CREB1 (**Figure 4B**). The luciferase reporter assay showed that miR-450a-5p mimic repressed the relative luciferase activities containing the WT 3’-UTR of CREB1, but had no obvious effect on Mut 3’-UTR of CREB1 (**Figure 4C**). Moreover, RT-qPCR and Western blot analysis were adapted to evaluate the expression of CREB1 in BGC-823 and SGC7901 cells transfected with miR-450a-5p mimic or NC, and the data revealed that up-regulation of miR-450a-5p decreased the expression of CREB1 (**Figures 4D, E**). These results indicated that miR-450a-5p negatively regulates CREB1.

### Knockdown of CREB1 Inhibits Gastric Cancer Cell Proliferation, Migration, Invasion, and Promotes Apoptosis

**Figure 5:**
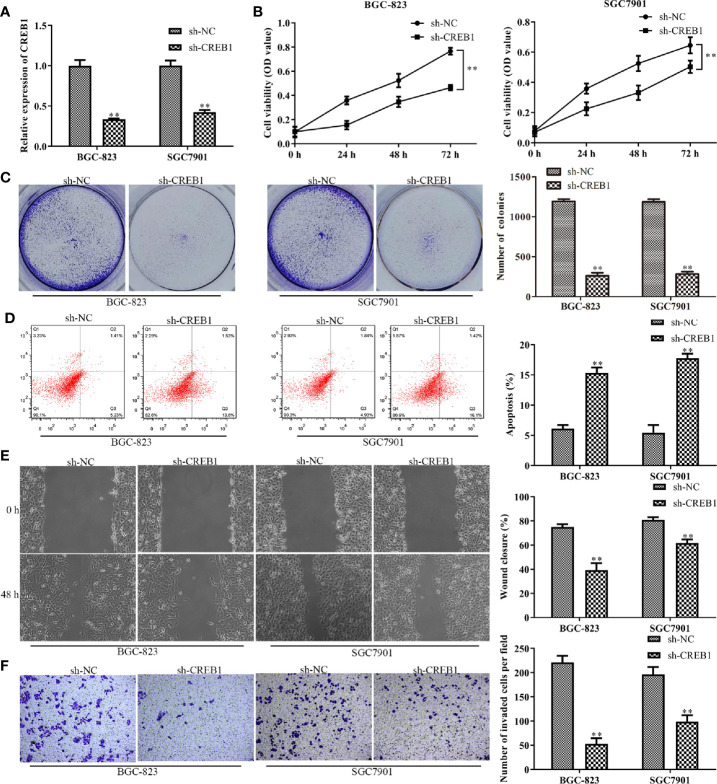
Knockdown of CREB1 affects gastric cancer cell proliferation, apoptosis, migration, and invasion. **(A)** RT-qPCR evaluated the efficiency of CREB1 knockdown in BGC-823 and SGC-7901 cells. **(B, C)** CCK-8 and colony formation assays on the cell proliferation of BGC-823 and SGC-7901 cells after knockdown of CREB1. **(D)** Flow cytometry on the cell apoptosis of BGC-823 and SGC-7901 cells after knockdown of CREB1. **(E, F)** Wound healing and transwell chamber assays on the cell migration and invasion of BGC-823 and SGC-7901 cells after knockdown of CREB1. ^**^ *P* <0.01 *vs.* sh-NC group.

The above results indicated that CREB1 is a target of miR-450a-5p in gastric cancer. We then examined whether CREB1 regulate the progression of gastric cancer. We knocked-down CREB1 in BGC-823 and SGC7901 cells and evaluated the knockdown efficiency by RT-qPCR analysis (**Figure 5A**). CCK-8 and colony formation assays were performed and the results showed that the cell viability and the number of colonies were significantly decreased in BGC-823 and SGC7901 cells transfected withsh-CREB1 compared with that in cells transfected with sh-NC (**Figures 5B, C**). Subsequently, flow cytometry was conducted to examine cell apoptosis after knocking-down of CREB1 expression in BGC-823 and SGC7901 cells. The results indicated that cell apoptosis was significantly increased in CREB1-knockdown BGC-823 and SGC7901 gastric cancer cells, compared with that in NC group (**Figure 5D**). Wound healing and transwell chamber assays showed that the gastric cancer cell migration and invasion capability was significantly inhibited in CREB1-knockdown BGC-823 and SGC7901 gastric cancer cells, compared with that in NC group (**Figures 5E, F**). These results showed that knockdown of CREB1 suppressed gastric cancer cell proliferation, migration, and invasion and induced apoptosis.

### CREB1 Overexpression Partially Restores the Effects of miR-450a-5p on Gastric Cancer

**Figure 6:**
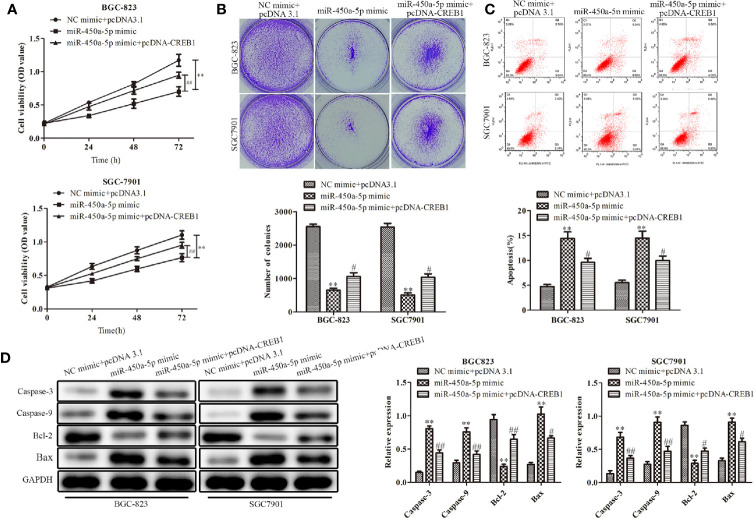
CREB1 overexpression partially restores the effects of miR-450a-5p on gastric cancer. **(A, B)** CCK-8 assay and colony formation assay on the cell proliferation of BGC-823 and SGC-7901 cells. **(C)** Flow cytometry on the cell apoptosis of BGC-823 and SGC-7901 cells. **(D)** Western blot analysis evaluated the expression of apoptosis-associated proteins in BGC-823 and SGC-7901 cells. ^**^ *P* <0.01 *vs.* NC + pcDNA3.1 group. ^#^ *P*<0.05, ^##^ *P* <0.05 *vs.* miR-450a-5p mimic group.

To further explore the role of CREB1 in mediating the inhibitory effect of miR-450a-5p on gastric cancer, BGC-823 and SGC7901 gastric cancer cells were overexpressed with CREB1 and co-transfected with miR-450a-5p mimic. CCK-8 and colony formation assays indicated that the cell viability and the number of colonies was significantly decreased in BGC-823 and SGC7901 cells in the miR-450a-5p mimic-treated group, compared with that in NC + pcDNA 3.1 group, suggesting that CREB partially abolished the inhibitory effects of miR-450a-5p mimic on cell proliferation of BGC-823 and SGC7901 gastric cancer cells (**Figures 6A, B**). Flow cytometric analysis indicated that miR-450a-5p mimic-induced promotion of cell apoptosis was prominently abrogated by CREB1 overexpression (**Figure 6C**).

At the molecular level, Western blot analysis indicated that the expression of apoptosis-associated proteins, including caspase-3, caspase-9, and Bax, were significantly increased in BGC-823 and SGC7901 gastric cancer cells treated with miR-450a-5p mimic, compared with that in NC + pcDNA 3.1 group, whereas the overexpression of CREB similarly abolished the activation effects of miR-450a-5p mimic on the expression of apoptosis-associated proteins, including caspase-3, caspase-9, and Bax. In contrast, the expression of anti-apoptotic protein of BCL-2 were decreased (**Figure 6D**). These results demonstrated that CREB1 overexpression partially restored the effects of miR-450a-5p on proliferation and apoptosis of gastric cancer.

### MiR-450a-5p Regulates Gastric Cancer Progression Through Inhibiting AKT/GSK-3β Signaling Pathway

**Figure 7:**
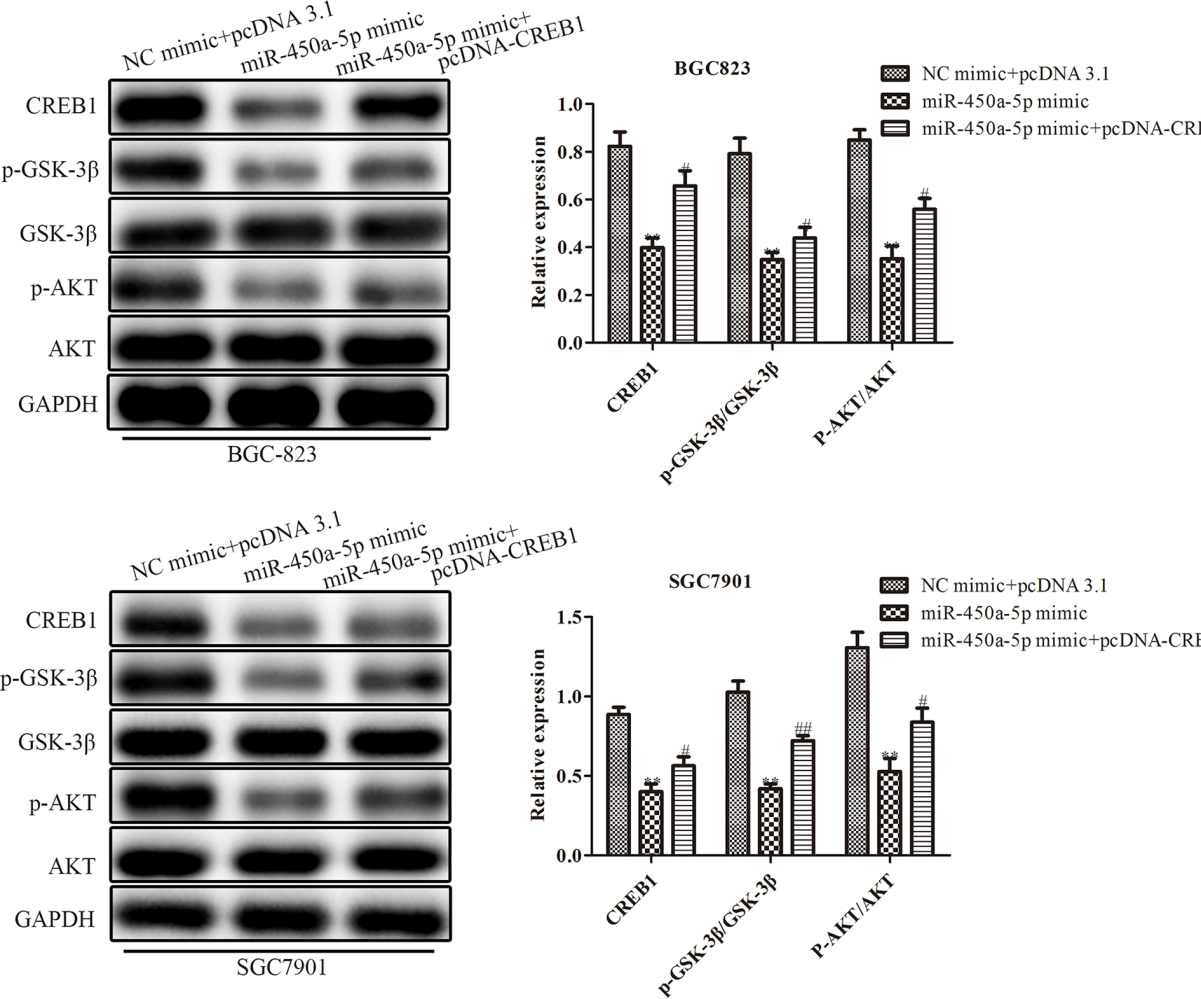
MiR-450a-5p regulates the AKT/GSK-3β signaling pathway. Western blotting evaluated the protein expression of CREB1, GSK-3β, AKT, p-GSK-3β, p-AKT. ^**^ *P* <0.05 *vs.* NC + pcDNA3.1 group. ^#^ *P*<0.05, ^##^ *P*<0.01 *vs.* miR-450a-5p mimic group.

**Figure 8:**
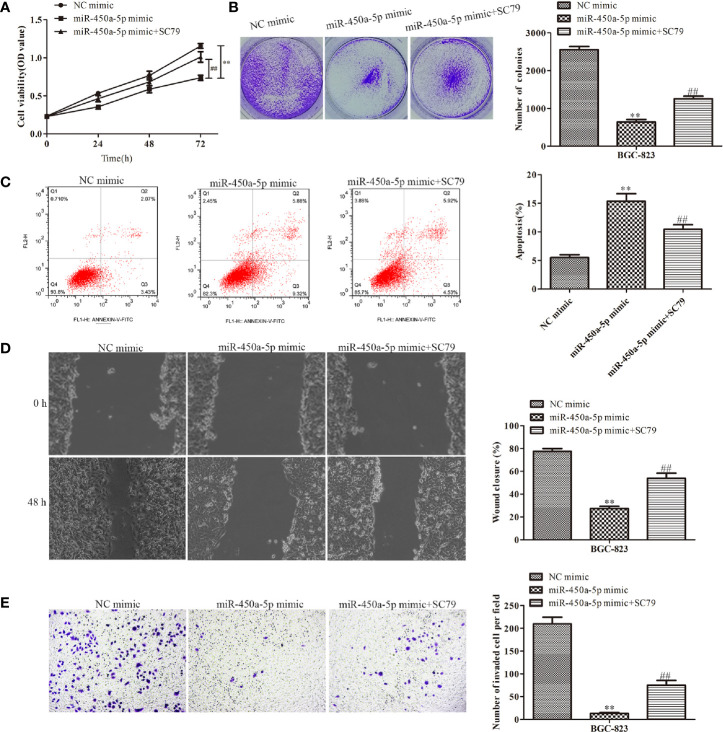
MiR-450a-5p inhibits the progression of gastric cancer by inhibiting the AKT/GSK-3β signaling pathway. **(A, B)** CCK-8 and colony formation assays on the cell proliferation of BGC-823 cells. **(C)** Flow cytometry on the cell apoptosis of BGC-823 cells. **(D)** Wound healing assay on the cell migration of BGC-823 cells after knockdown of CREB1. **(E)** Transwell chamber assay on the cell invasion of BGC-823 cells after knockdown of CREB1. ^**^ *P* <0.01 *vs.* NC mimic group. ^##^ *P* <0.01 *vs.* miR-450a-5p mimic group.

Given the importance of AKT/GSK-3β in the regulation of gastric cancer, we investigated the expression of proteins in the AKT/GSK-3β signaling pathway by Western blot assay (**Figure 7**). We found that the expression of the AKT/GSK-3β pathway-associated protein, including GSK-3β and AKT, had no significantly change. However, the phosphorylated proteins in this pathway were markedly decreased in response to the overexpression of miR-450a-5p, while CREB overexpression increased AKT/GSK-3β phosphorylated proteins in BGC-823 cells treated with miR-450a-5p mimic. To further identify the role of AKT/GSK-3β in mediating the anti-gastric cancer role of miR-450a-5p, we use SC79 as an activator of the AKT/GSK-3β signaling pathway. As shown in **Figures 8A, B, D, E**, CCK-8, colony formation, wound healing, and transwell chamber assays showed that the gastric cancer cell proliferation, migration, and invasion abilities were significantly decreased in the miR-450a-5p mimic-treated group, while significantly increased in themiR-450a-5p mimic + SC79 group compared with that in miR-450a-5p mimic group. These results were further confirmed by flow cytometry, which showed that the apoptosis rate markedly increased in miR-450a-5p mimic group, while significantly decreased in miR-450a-5p mimic + SC79 group (**Figure 8C**). These results suggested that miR-450a-5p represses gastric cancer progression by targeting CREB1 through inhibiting AKT/GSK-3β signaling pathway.

## Discussion

Over the past years, more and more evidence indicated that microRNAs are involved in regulating various biological and pathological processes. In this study, we found that miR-450a-5p was significantly downregulated in gastric cancer cells. Gain-of-function analysis indicated that overexpression of miR-450a-5p inhibited cell proliferation, migration, and invasion, and facilitated cell apoptosis in gastric cancer cells *in vitro*, and suppressed tumor growth *in vivo*. Moreover, we found that overexpression of miR-450a-5p increased the expression of apoptosis-associated proteins, including caspase-3, caspase-9, and Bax, while inhibited the expression of anti-apoptotic protein Bcl-2. In addition, overexpression of miR-450a-5p suppressed the expression of matrix protein enzymes MMP-2 and MMP-9, and promoted cancer cell migration and invasion. Our results suggested that miR-450a-5p could function as a tumor suppressor to inhibit gastric cancer cell growth. Although the public data shows miR-450a-5p has a bit higher expression in gastric tumors (**Supplementary Data**), the role of miR-450a-5p in clinic tissues still need further study.

A number of studies have been focused on trying to identify effective new targets for the treatment of gastric cancer. MiRNAs have been attracted great attention. Various miRNAs were found to be associated with cancer development and progression, and therefore, they could be potential molecular targets for cancer treatment. In the area of gastric cancer, Feng et al. reported that miR-518 suppresses the progression of gastric cancer by promoting cell apoptosis *via* targeting MDM2 (19). Therefore, miR-518 may be a promising therapeutic target for gastric cancer. Liu et al. demonstrated that miR-204 modulates the EMT process to inhibit gastric cancer cell migration and invasion *via* regulating snai1both *in vitro* and *in vivo* (20). Wang et al. confirmed that miR-129-5p inhibits gastric cancer cell proliferation and EMT by high-mobility group box 1 (HMGB1), and could be a potential target for the treatment of gastric cancer (21). Wang et al. showed that overexpression of miR-128b inhibits cell proliferation, migration, and invasion, and promotes apoptosis in gastric cancer cells *via* down-regulating adenosine 2b receptor (A2bR) (22). Wu et al. reported that up-regulation of miR-449c suppresses gastric cancer cell growth and promotes apoptosis, while down-regulation of miR-449c promotes gastric cancer cell growth and inhibits apoptosis (23). Wang et al demonstrated that over-expression of miR-217 inhibits gastric cancer cell proliferation, invasion and promotes apoptosis *via* regulating geriatric palliative care 5 (GPC5), and could be a potential therapeutic target (24). In addition to the above mentioned miRNAs, other miRNAs, for instance, miR-376c-3p (25), miR-133b (26), miR-99b-5p and miR-203a-3p (27), miR-491-5p (28), miR-143 (29) are involved in the regulation of the development and progression of gastric cancer. Our study suggested that miR-450a-5p plays critical roles in inhibiting the development and progression of gastric cancer.

Multiple lines of evidence have proofed that miRNAs exert their diverse biological functions, such as acting as key signal transduction mediators and regulating cell activities, mainly by degrading mRNA or suppressing mRNA translation. Therefore, we further explored the potential downstream target of miR-450a-5p by bioinformatic analysis. TargetScan revealed that CREB1 could be a potential candidate of miR-450a-5p. Dual luciferase reporter assay, RT-qPCR and Western blot analysis confirmed that CREB1 is negatively regulated by miR-450a-5p. Therefore, miR-450a-5p might alleviate the development and progression of gastric cancer *via* targeting CREB1. In this study, we also found that CREB overexpression increased the phosphorylated proteins of AKT/GSK-3β signaling pathway, promoting cell proliferation, migration, and invasion, and suppressed apoptosis. Simultaneously, knockdown of CREB1 suppressed gastric cancer cell growth.

It was well known that the AKT/GSK-3βsignaling pathway is closely associated with cancer development and progression. Hua et al. reported that knockdown of ANXA11 suppressed gastric cancer cell proliferation, invasion, and migration through the AKT/GSK-3β signaling pathway (30). Pan et al. demonstrated that CD36 regulated palpitate acid-induced metastasis in gastric cancer by AKT/GSK-3β/β-catenin signaling pathway (31). Targeting AKT/GSK-3β signaling pathway might be critical for cancer intervention. In our study, we found that miR-450a-5p blocked the AKT/GSK-3β signaling pathway to inhibit the growth of gastric cancer cells and the level of miR-450a-5p were related to the inhibitory ability of the AKT/GSK-3β signaling pathway.

Previous study reported that miR-21, miR-450 and miR-149 participate in the regulation of cancers (13, 15). Moreover, miR-21 and miR-149 are associated with the development of gastric cancer (12, 14). As miR-21 and miR-149 play a role in other cancers, for instance, colorectal cancer (32), ovarian cancer (33), breast cancer (34), bladder cancer (35), and hepatocellular carcinoma (36), it is worthy to investigate whether miR-450a-5p also plays a role in these cancers. Future study will be directed to understand the role of miR-450a-5p in various cancers, which will provide the advantages of using miR-450a-5p as a molecular therapeutic target.

In conclusion, miR-450a-5p targets CREB1 and inhibits the AKT/GSK-3β signaling pathway to repress the development and progression of gastric cancer. Our study suggests that miR-450a-5p is a tumor suppressor and could be a new molecular target for the treatment of gastric cancer.

## Data Availability Statement

The raw data supporting the conclusions of this article will be made available by the authors, without undue reservation.

## Ethics Statement

The animal study was reviewed and approved by Use Committee and approved by the Ethics Committee of the First Affiliated Hospital of USTC.

## Author Contributions

X-YH conceived the study. Y-JZ and X-YH designed the study. JZ, Y-CW, and LW performed the literature search and data extraction. Y-JZ and X-YH drafted the manuscript. All authors contributed to the article and approved the submitted version.

## Funding

This work was supported by the Youth Fund Project of The First Hospital of USTC (West District, No. 2018YJQN002).

## Conflict of Interest

The authors declare that the research was conducted in the absence of any commercial or financial relationships that could be construed as a potential conflict of interest.
