# Supplementary material for: MiR-450a-5p Inhibits Gastric Cancer Cell Proliferation, Migration, and Invasion and Promotes Apoptosis via Targeting CREB1 and Inhibiting AKT/GSK-3β Signaling Pathway
Source: Front Oncol. 2021 Mar 29;11:633366. doi: 10.3389/fonc.2021.633366 (PMC8039465; doi:10.3389/fonc.2021.633366)
Supplement: Supplementary file 1 [file DataSheet_1.pdf]

Figure 1F

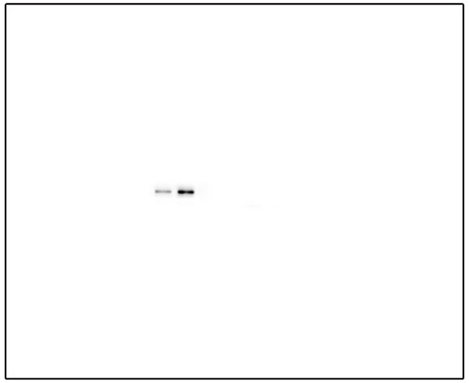

Cleaved Caspase9-35kDa

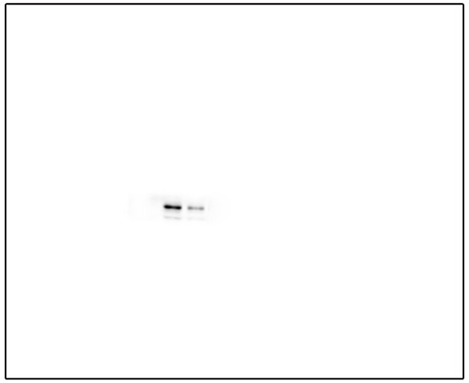

Bcl2-26kDa

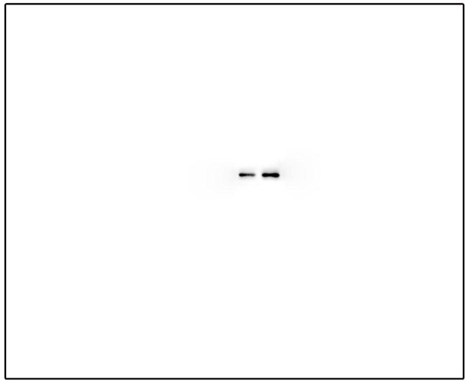

Cleaved Caspase9-35kDa

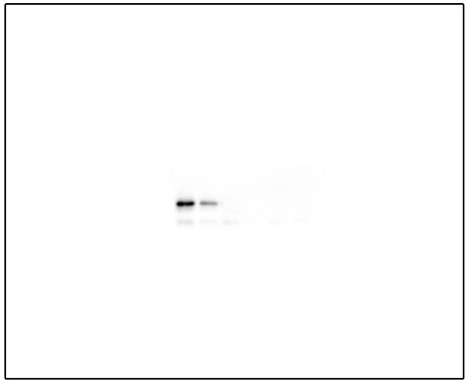

Bcl2-26kDa

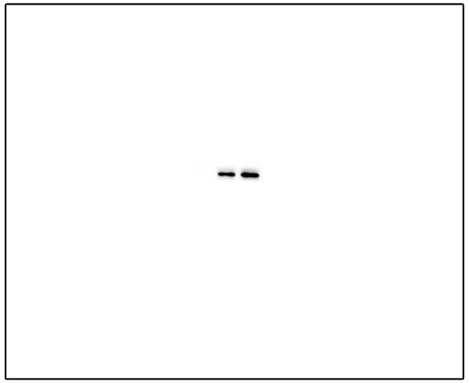

Cleaved caspase3-17kDa

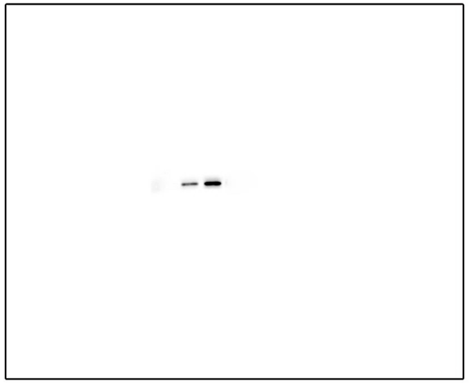

Bax-21kDa

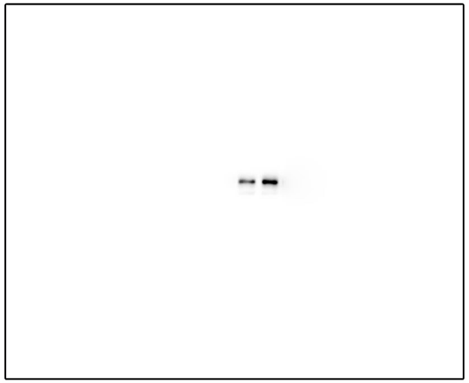

Cleaved caspase3-17kDa

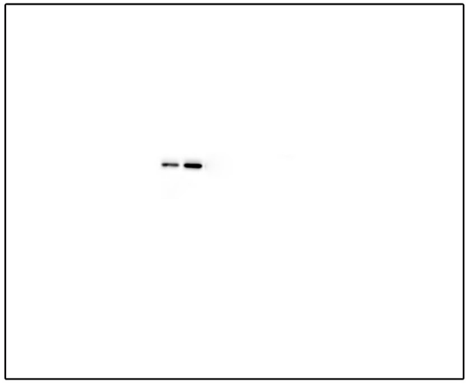

Bax-21kDa

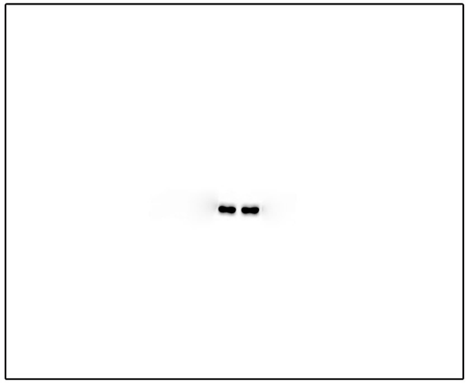

GAPDH 37kDa

BGC823

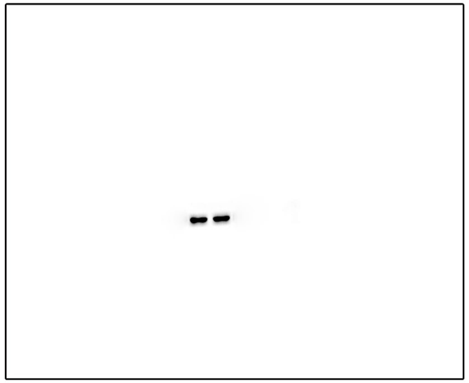

GAPDH 37kDa

SGC7901

Figure 2E

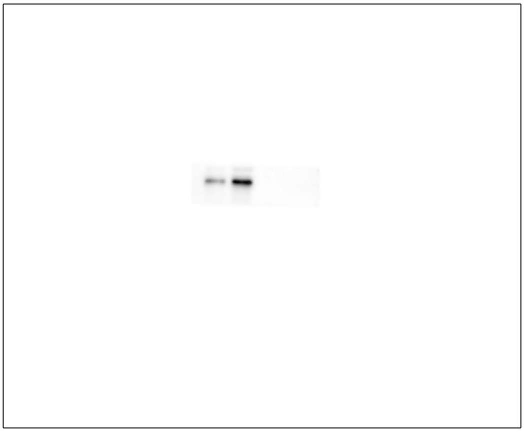

Cleaved-caspase3 17kDa

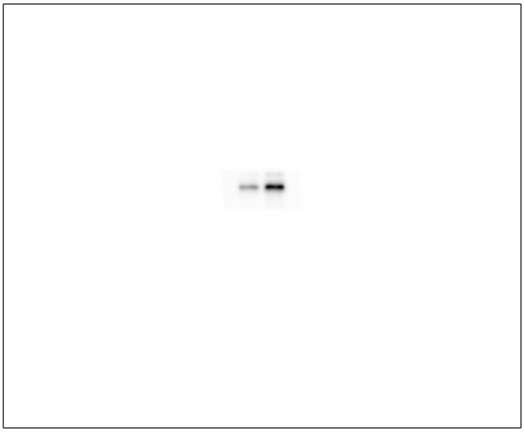

Cleaved-caspase9 35kDa

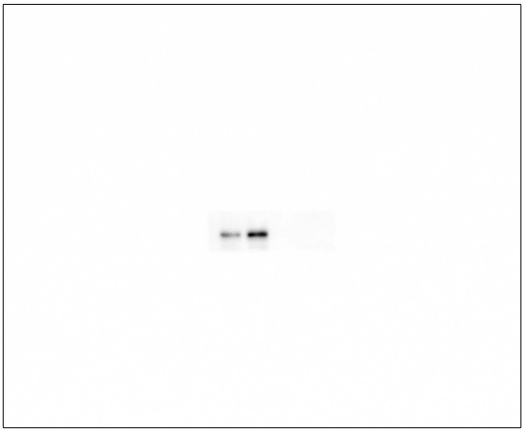

Bax 21kDa

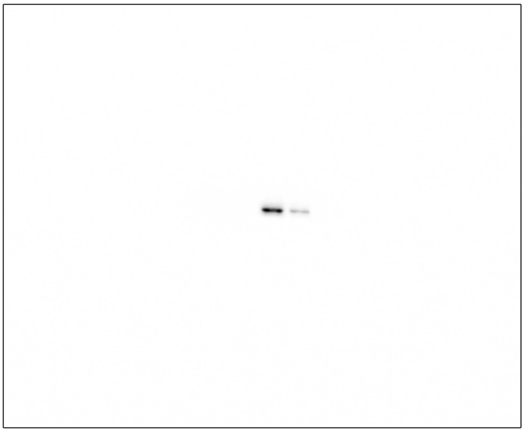

Bcl2 26kDa

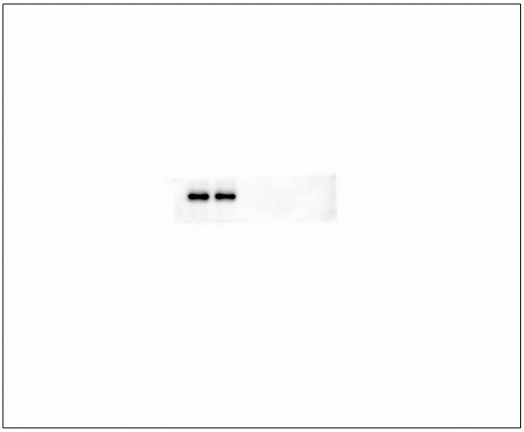

GAPDH 37kDa

Figure 3C

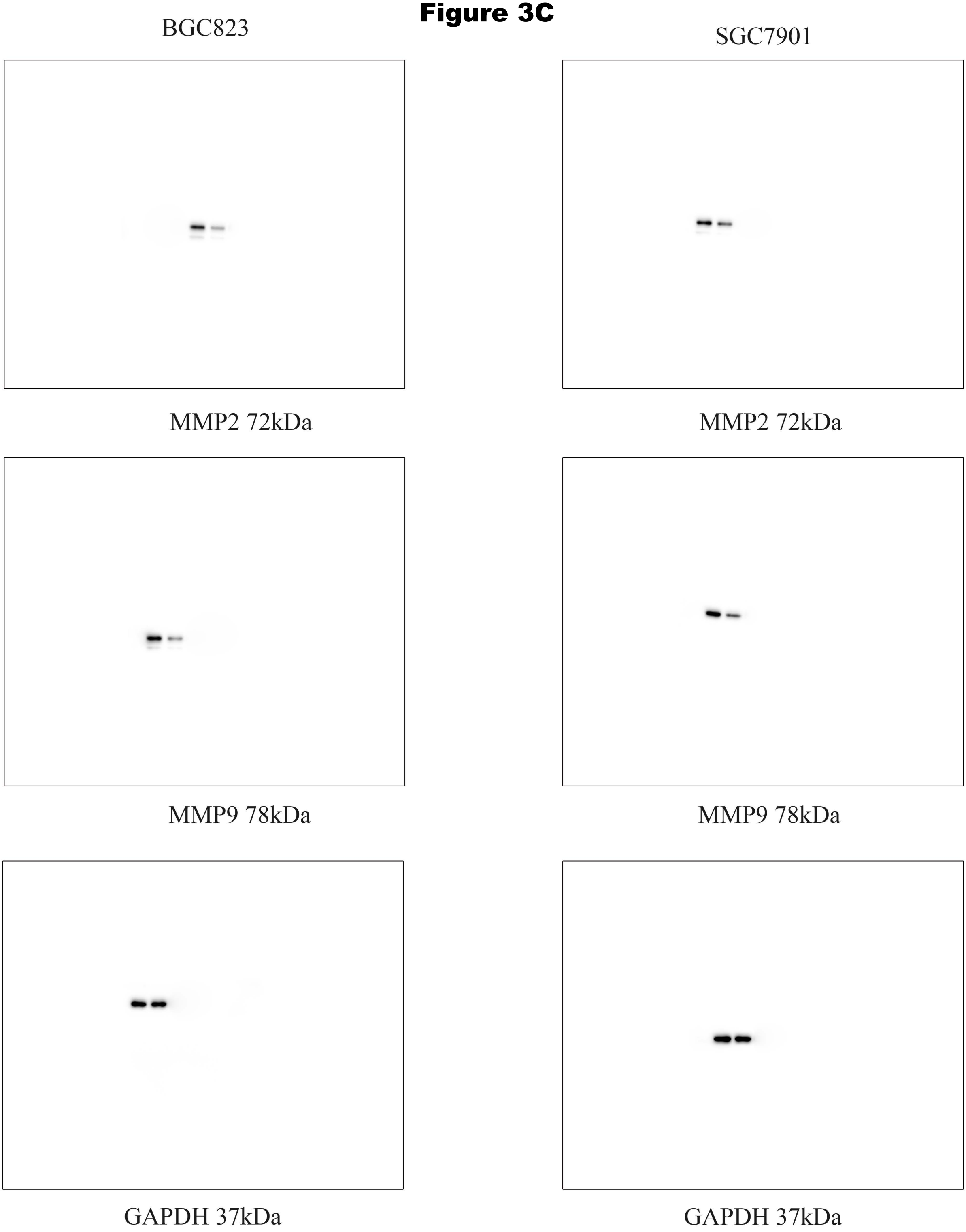
BGC823
SGC7901
MMP2 72kDa
MMP2 72kDa
MMP9 78kDa
MMP9 78kDa
GAPDH 37kDa
GAPDH 37kDa

Figure 4E

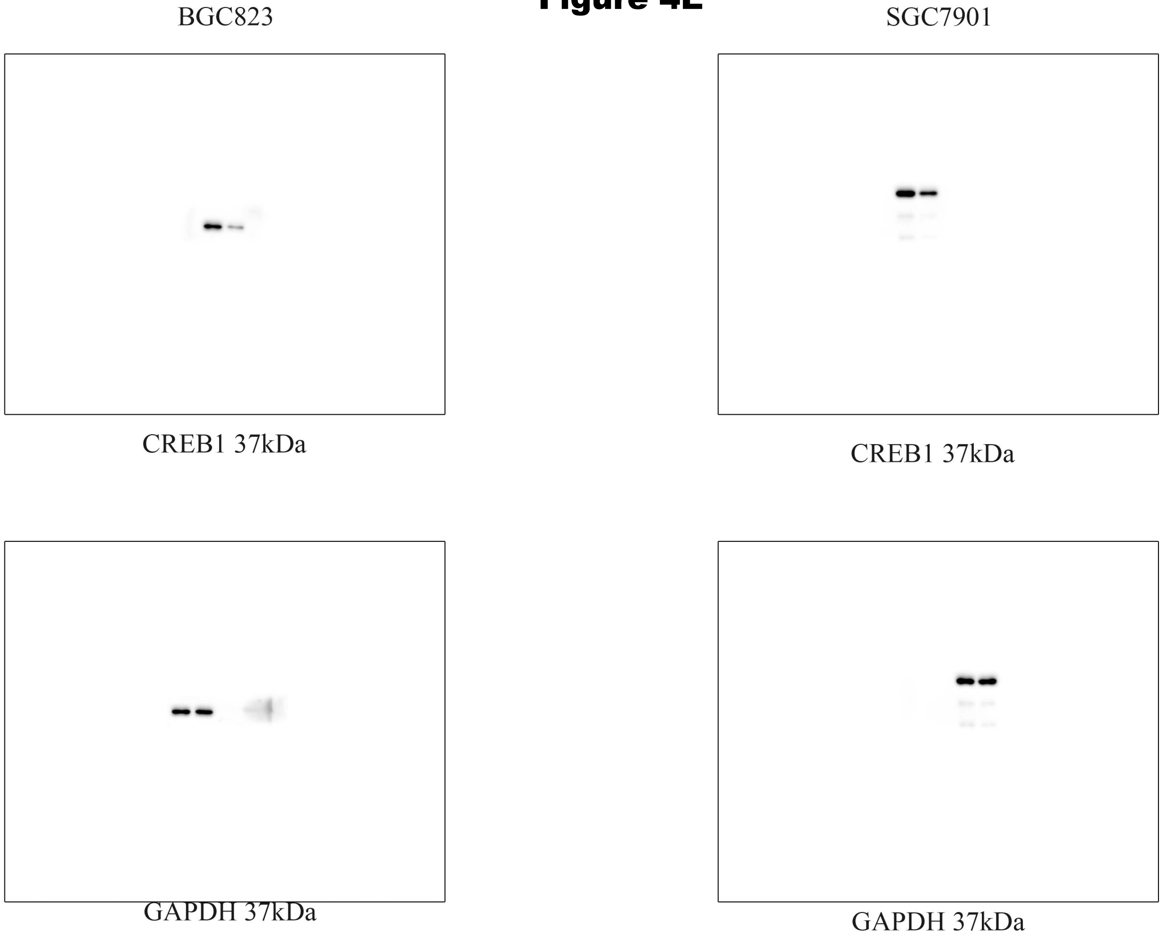
BGC823
SGC7901
CREB1 37kDa
CREB1 37kDa
GAPDH 37kDa
GAPDH 37kDa

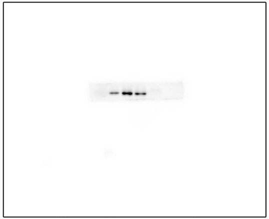

Cleaved Caspase9-35kDa

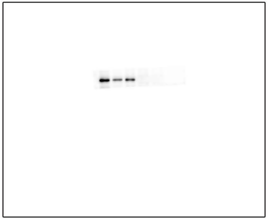

Bcl2-26kDa

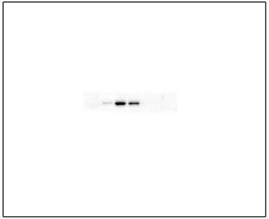

Cleaved Caspase9-35kDa

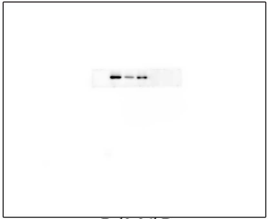

Bcl2-26kDa

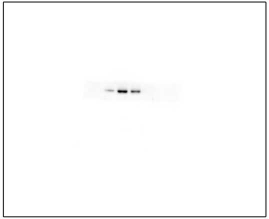

Cleaved caspase3-17kDa

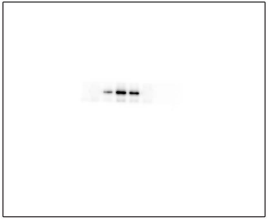

Bax-21kDa

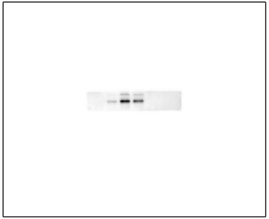

Cleaved caspase3-17kDa

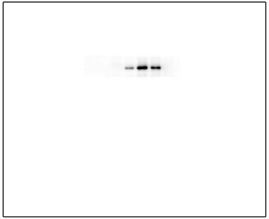

Bax-21kDa

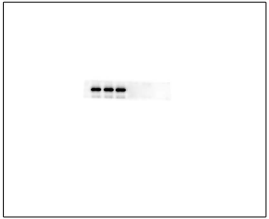

GAPDH 37kDa

BGC823

Figure 6D

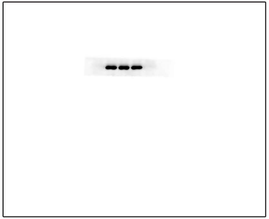

GAPDH 37kDa

SGC7901

Figure 7

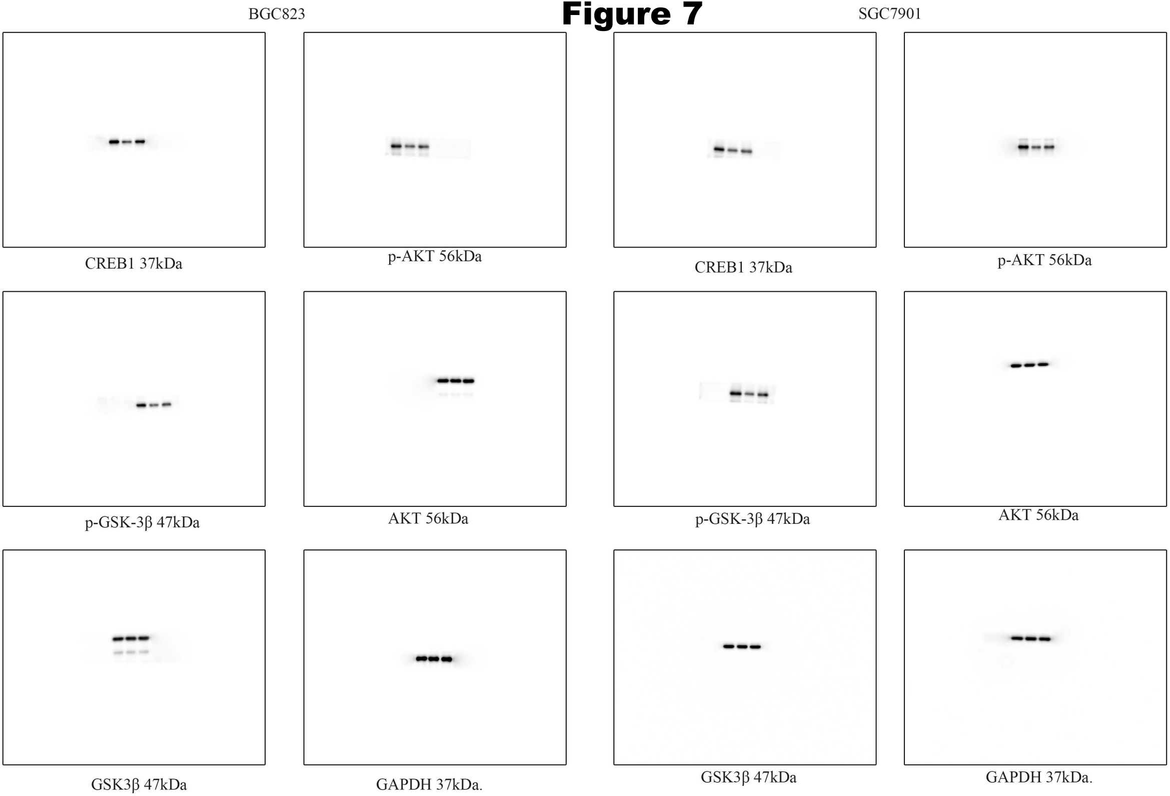
BGC823
SGC7901
CREB1 37kDa
p-AKT 56kDa
CREB1 37kDa
p-AKT 56kDa
p-GSK-3β 47kDa
AKT 56kDa
p-GSK-3β 47kDa
AKT 56kDa
GSK3β 47kDa
GAPDH 37kDa.
GSK3β 47kDa
GAPDH 37kDa.
