# Supplementary figures and images for: MiR-450a-5p Inhibits Gastric Cancer Cell Proliferation, Migration, and Invasion and Promotes Apoptosis via Targeting CREB1 and Inhibiting AKT/GSK-3β Signaling Pathway
Source: Front Oncol. 2021 Mar 29;11:633366. doi: 10.3389/fonc.2021.633366 (PMC8039465; doi:10.3389/fonc.2021.633366)

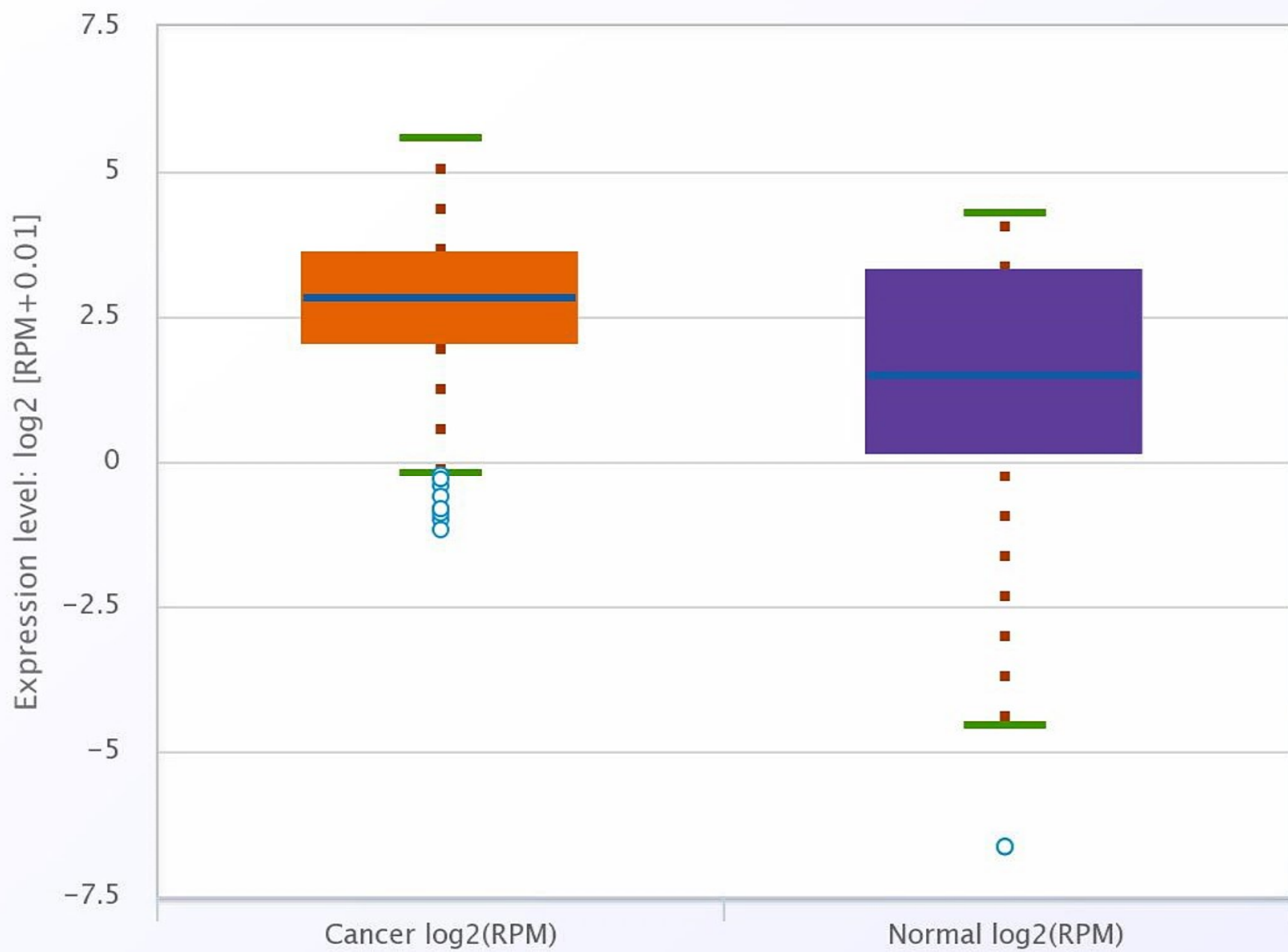

Supplement: Supplementary file 2 [file DataSheet_2.pdf]
